# Hanging Position of Artificial Nest Boxes Affects Reproductive Success of Russet Sparrow *Passer cinnamomeus*

**DOI:** 10.3390/ani15101413

**Published:** 2025-05-14

**Authors:** Pan Peng, Wanzhu Chen, Nursoltan Qianhan, Hang Yu, Qian Hu, Jiagui Zhu, Hua Huang, Zhiyong Du, Jianqiang Li

**Affiliations:** 1 School of Ecology and Nature Conservation, Beijing Forestry University, Beijing 100083, China; pengpan@bjfu.edu.cn (P.P.); chenwanzhu410@bjfu.edu.cn (W.C.); newuncia@163.com (N.Q.); yuhang94@bjfu.edu.cn (H.Y.); huqian1995@bjfu.edu.cn (Q.H.); 2 Dongzhai National Nature Reserve Affairs Center, Luoshan, Xinyang 464236, China; dzzjg_2022@163.com (J.Z.); zwp7054@163.com (H.H.); dzy0376@sina.com (Z.D.)

**Keywords:** artificial nest box, russet sparrow, nest location, reproductive success

## Abstract

Secondary cavity-nesting birds can utilize artificial nest boxes provided by humans for breeding, making nest boxes important approaches for attracting birds. Artificial nest boxes are therefore widely used in fields such as ornithological research and avian diversity conservation. Under natural conditions, the location of nests often affects the reproductive success of birds. Therefore, understanding the impact of nest box hanging positions on the breeding success of secondary cavity-nesting birds is of great significance for guiding the placement of artificial nest boxes. In this study, we explored the potential factors, including nest-box location, that affect the reproductive success of the russet sparrow (*Passer cinnamomeus*) by monitoring the nest-site characteristics and reproductive parameters such as clutch size and brood size of those breeding in artificial nest boxes near Dongzhai National Nature Reserve, Henan, China, from April to July 2024. Russet sparrow nesting in boxes on coniferous trees and utility poles showed no significant differences in clutch size, hatching rate, brood size, pre-laying nest-abandonment rate, and whether the breeding was ultimately successful. Nests with earlier egg-laying dates had larger clutch sizes, higher hatching rates, and greater brood sizes. However, the number of fledglings and fledging success were not affected by laying date, and fledging success was not affected by either the distance from the nest site to the nearest road, or the brood size. Both larger brood size and greater distance from the nearest road were associated with increased numbers of fledglings. Furthermore, russet sparrows nesting in boxes on coniferous trees produced significantly more fledglings and exhibited higher fledging success compared to those nesting on utility poles, suggesting that nest boxes’ hanging position may influence russet sparrow reproductive success. These findings not only supplement existing ecological data on russet sparrow reproduction in artificial nest boxes but also provide important insights for avian conservation practices, highlighting the necessity of fully considering hanging position when deploying artificial nest boxes for birds.

## Simple Summary

In the fields of ornithological research and bird conservation practices, artificial nest boxes are widely used as an important tool. However, there are still many uncertainties regarding the impact of the hanging positions of artificial nest boxes on the reproductive success of birds. To explore the relationship between factors such as the hanging positions of artificial nest boxes and the reproduction of the russet sparrow (*Passer cinnamomeus*), we recorded the breeding parameters and nest-site characteristics of the russet sparrow. The results showed that the number of fledglings and fledging success of russet sparrow breeding in nest boxes on coniferous trees were higher than those breeding in nest boxes on utility poles. Also, the further the nest box was from the nearest road, the more fledglings came from the nest. This suggests that the hanging positions of nest boxes may affect the reproductive success of the russet sparrow, providing invaluable implications for using nest boxes in bird population management.

## 1. Introduction

Reproduction is a vital biological characteristic for avian species to propagate their offspring and constitutes a crucial phase in avian life history [1], playing an indispensable role in the perpetuation of bird populations [2]. Consequently, avian reproductive ecology has always been a subject of extensive attention and research. Conducting research on avian reproductive ecology is not merely an observation of reproductive behaviors, but more importantly, it may elucidate the impacts of ecological factors on reproductive strategies, thereby providing insights into population dynamics and adaptive evolution of bird species [3].

Nest site selection is an essential component of avian reproduction, a behavioral strategy that has been shaped through their evolutionary history [4]. In the wild, nest-site selection is influenced by a variety of factors, including predation risk [5], interspecific and intraspecific competition [6], food availability [7], social information [8], and natal experience [9]. Therefore, high-quality nest sites can minimize the impact of predators and other factors such as competition, thereby enhancing reproductive success and fitness [10].

Secondary cavity-nesting birds are capable of utilizing artificial nest boxes provided by humans for breeding. Artificial nest boxes have been widely employed in various fields, such as ornithological research and avian biodiversity conservation [11,12,13,14]. However, the hanging position of nest boxes may exert differential impacts on avian reproduction. For example, studies have found that the fledging success of the European roller (*Coracias garrulus*) breeding in artificial nest boxes is higher in areas with more surrounding arable land [15], while southern ground hornbills (*Bucorvus leadbeateri*) breeding in higher nest boxes exhibited greater reproductive success [16]. Therefore, understanding how the hanging position of nest boxes influences the reproductive success of secondary cavity-nesting birds holds substantial significance for guiding the optimal placement of artificial nest boxes. Moreover, artificial nest boxes also have their drawbacks and some researchers have pointed out the negative impacts they can have on birds [17]. Nevertheless, despite these known effects of artificial nest boxes on birds, research on this topic remains inadequate and warrants further investigations.

The russet sparrow (*Passer cinnamomeus*), a species within the family Passeridae of the order Passeriformes, has a wide distribution in China [18]. It is a secondary cavity-nesting bird species that typically breeds in locations such as tree cavities and wall holes. The present study investigated the reproductive ecology of russet sparrows breeding in artificial nest boxes, and explored the relationship between nest box hanging position and reproductive success to gain a deeper understanding of the impact of artificial nest boxes on avian reproduction.

## 2. Materials and Methods

### 2.1. Study Area and Nest Box Installation

The study area, which was approximately 14 km^2^, was located in Pengzhuang and Yuanchong villages, Lingshan Town, Luoshan County, Henan Province (31°59′ N, 114°21′ E). The region is situated near the Dongzhai National Nature Reserve, characterized by a mild and humid climate as well as a hilly and mountainous topography, and is rich in flora and fauna [19]. The dominant vegetation consists of tree species such as *Pinus massoniana*, *Cunninghamia lanceolata*, *Liquidambar formosana*, and *Pterocarya stenoptera*. Additionally, the area supports a diverse shrub community, including *Rhododendron simsii*, *Lindera glauca*, *Ilex cornuta*, and *Camellia oleifera*. The region also features agricultural land uses such as paddy fields and tea gardens. The russet sparrow is a common resident bird in this area.

In the breeding season in 2024, a total of 537 artificial nest boxes have been installed in this area to attract cavity-nesting bird species such as the russet sparrow and the Japanese tit (*Parus minor*). Nest boxes were typically hung on trees at the forest edge or on utility poles along roadsides, with a minimum distance of 30 m between adjacent nest boxes [19]. The artificial nest boxes used in this study comprised wooden nest boxes (*n* = 88) and polyvinyl chloride (PVC) nest boxes (*n* = 449). The wooden nest boxes were rectangular in shape, with a square base measuring 14 cm on each side. The PVC nest boxes were cylindrical, with a base diameter of 16 cm. Both types of nest boxes had a height of 26 cm and an entrance hole diameter of 3.5 cm.

### 2.2. Monitoring of the Breeding Habits of the Russet Sparrow

From April to July 2024, all nest boxes within the study area were checked at biweekly intervals to promptly determine the utilization of nest boxes by the russet sparrow. When nest materials of the russet sparrow, mainly consisting of pine needles, dry grass, and feathers, were found in the nest boxes, the nests were checked every 3–4 days to confirm reproductive parameters such as the date of egg-laying, clutch size, brood size, and the number of fledglings. Additionally, the positions where the nest boxes were hung (i.e., tree species or utility poles) were recorded.

### 2.3. Quantification of the Distance from Nest Boxes to the Nearest Road

The nearest distance of nest boxes to roads was quantified using ArcGIS 10.8 software. Based on the World Imagery remote sensing imagery acquired on 15 August 2024 (with a resolution of 1:3000), the study area’s rural and paved roads (excluding all pedestrian footpaths, temporary research access routes, and non-vehicular pathways) were delineated at a scale of 1:600. The geographic distance (in meters) between each nest box and the nearest road was calculated using the proximity analysis tool in ArcGIS 10.8 software. This analysis aimed to assess the potential impact of road proximity on the reproductive success of the russet sparrow.

### 2.4. Data Analysis

All analyses were conducted using R version 4.4.2 [20]. In this study, only three nests of russet sparrows breeding in nest boxes hung on broadleaf trees and five nests in wooden nest boxes were recorded. Except for being included when reporting basic reproductive parameters, these nests were excluded from the analyses of factors influencing reproductive success due to the small sample size.

We used chi-squared tests to analyze whether there were significant differences in the pre-laying nest abandonment rate and reproductive success between nests of the russet sparrow in nest boxes on coniferous trees and those on utility poles. A nest was treated as a successful nest if at least one nestling fledged; otherwise, the nest was considered to have failed in reproduction. When the expected counts in the data were less than 5, a chi-squared test with Monte Carlo simulation was employed for analysis.

The remaining analyses were conducted using a linear mixed model (LMM) or generalized linear mixed model (GLMM). To analyze the factors influencing clutch size, a LMM was constructed with clutch size as the response variable. The explanatory model terms included the distance of the nest box to the nearest road and its quadratic term (implemented using the poly function in R), relative laying date (the number of days between the nest’s date of laying the first egg and the earliest observed laying date in the population), and the hanging position of the nest box (coniferous tree or utility pole, treated as a categorical variable) as well as the interactive effects between the hanging position of the nest box and each of the remaining variables. Nest box ID was included as a random effect to account for the non-independence of data from individuals using the same nest box for breeding.

To analyze the factors influencing brood size and hatching success (brood size divided by clutch size), separate models were constructed with brood size and hatching success as response variables, using a LMM and a GLMM, respectively. The explanatory model terms included the distance of the nest box to the nearest road and its quadratic term (implemented using the poly function in R), clutch size, relative laying date, and the hanging position of the nest box, as well as the interactive effects between the hanging position of the nest box and each of the remaining variables. Nest box ID was set as a random effect.

To analyze the factors influencing the number of fledglings, a LMM was constructed with the number of fledglings as the response variable. The explanatory model terms included the distance of the nest boxes to the nearest road and its quadratic term (implemented using the poly function), brood size, relative laying date, and the hanging position of the nest boxes as well as the interactive effects between the hanging position of the nest box and each of the other variables. Nest box ID was set as a random effect.

To analyze the factors influencing fledging success (number of fledglings divided by brood size), a GLMM was constructed with fledging success as the response variable. The explanatory model terms included brood size, relative laying date, and the hanging position of the nest boxes, as well as the interactive effects between the hanging position of the nest box and each of the other variables. Nest box ID was set as a random effect.

The aforementioned LMMs and GLMMs were constructed using the buildglmmTMB function from the buildmer package (version 2.11) [21] in R. Based on the initial explanatory variables of the aforementioned models, the optimal models containing only significant independent variables were derived through backward stepwise regression and likelihood ratio tests. In addition to the aforementioned analyses, the car package (version 3.1-3) [22] in R was utilized to conduct a multicollinearity test on the independent variables of the models prior to analysis. The multicollinearity among the independent variables was found to be low (VIF < 2), indicating limited collinearity issues [23].

Statistical significance was considered at *p* < 0.05. Unless otherwise specified, data presented in the results are expressed as mean ± SD.

## 3. Results

### 3.1. The Breeding Habits of the Russet Sparrow

**Figure 1:**
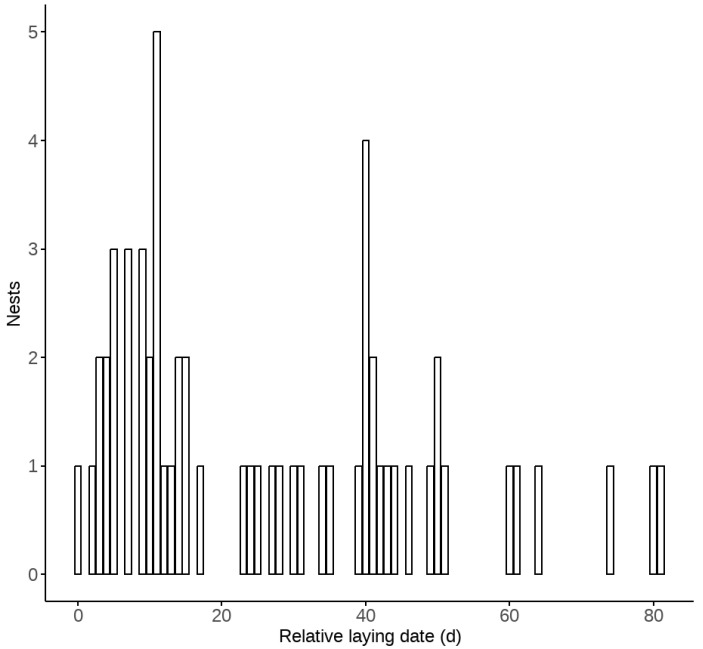
The number of egg-laying nests in different time periods (the relative laying date of 0 day was 17 April).

A total of 54 nest boxes were found to have been utilized by russet sparrows, with a total of 65 breeding attempts recorded. Among them, 32 nest boxes were hung on coniferous trees, 19 were hung on utility poles, and 3 were hung on broad-leaved trees. The russet sparrow lays one egg per day, with the earliest recorded laying date being 17 April and the latest being 7 July. The peak egg-laying period occurred in late April and mid-to-late May (Figure 1). The average clutch size was 4.61 ± 0.58 eggs (*n* = 64 nests). The average incubation period was 11.38 ± 1.24 days (*n* = 18 nests). The average brood size was 3.93 ± 0.94 chicks (*n* = 57 nests). The average hatching rate was 0.86 ± 0.18 (*n* = 57 nests). The average number of fledglings was 3.68 ± 1.09 chicks (*n* = 44 nests), and average fledging success was 0.93 ± 0.18 (*n* = 44 nests) (Table 1).

### 3.2. The Impact of Nest-Site Selection on the Breeding Habits of the Russet Sparrow

**Figure 2:**
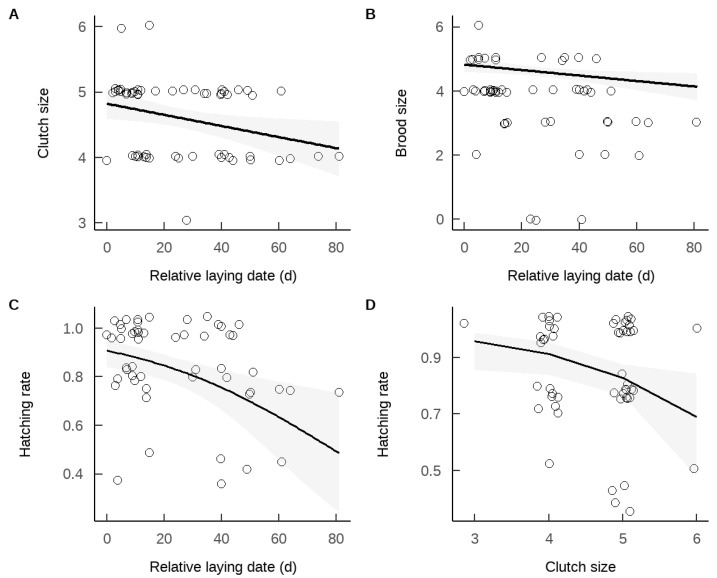
The relationships between relative laying date and clutch size (**A**), brood size (**B**), and hatching rate (**C**), and the relationship between clutch size and hatching rate (**D**) of *Passer cinnamomeus*. The points in the figure represent the original data after jittering. The line (with the shaded area indicating the 95% confidence interval) is fitted by the predicted values of the best model.

The analysis revealed that there were no significant differences in pre-laying nest abandonment rate (χ^2^ = 0.754, *p* = 0.634) or final reproductive success (χ^2^ = 1.008, df = 1, *p* = 0.315) between russet sparrows breeding in nest boxes on coniferous trees and those nesting on utility poles. The distance of nest boxes to the nearest road and the tree species on which nest boxes were hung did not significantly influence the clutch size of the russet sparrow (Table 2). However, later laying dates were associated with smaller clutch sizes (Table 2 and Figure 2A).

The brood size of the russet sparrow was not significantly influenced by the distance of nest boxes to the nearest road, the hanging position of the nest box, or clutch size (Table 2). However, earlier laying dates were associated with larger brood sizes (Table 2 and Figure 2B).

The hatching rate of the russet sparrow was not significantly influenced by the distance of nest boxes to the nearest road or the hanging position of the nest box (Table 2). However, relative laying date (Table 2 and Figure 2C) and clutch size (Table 2 and Figure 2D) significantly affected the hatching rate, with fewer clutch sizes and earlier laying dates associated with higher hatching rate.

**Figure 3:**
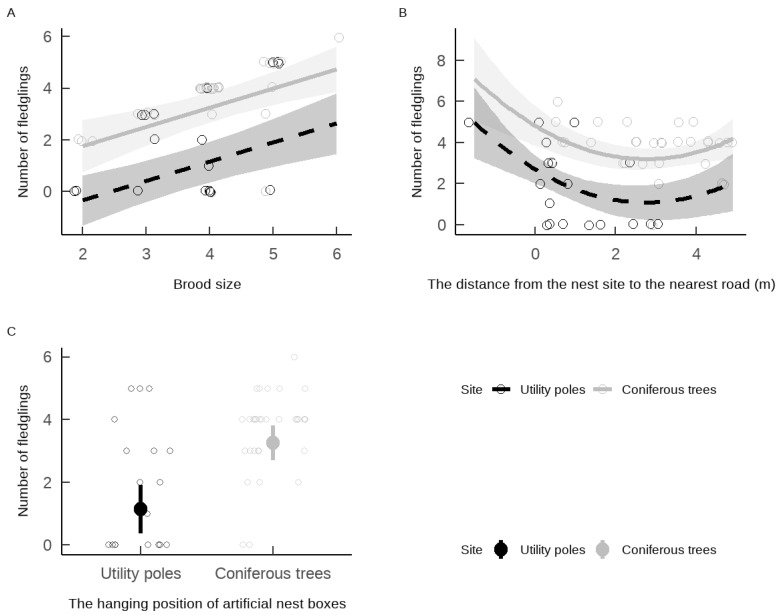
The effect of brood size (**A**), the distance from the nest site to the nearest road (**B**), and the hanging position of artificial nest boxes (**C**) on the number of fledglings of *Passer cinnamomeus*. The points in the figure represent the original data after jittering. The line (with the shaded area being the 95% confidence interval) is fitted by the predicted values of the best model. The solid circles and vertical lines in (**C**) represent the average number of fledglings and the 95% confidence interval of this type of tree species.

The number of fledglings from russet sparrow nests was not significantly influenced by laying date but was significantly affected by the distance of nest boxes to the nearest road, brood size, and the hanging position of the nest boxes (Table 2). Specifically, nests with larger brood sizes and those located farther from the nearest road produced more fledglings (Table 2, Figure 3A,B). Additionally, russet sparrows breeding in nest boxes on coniferous trees had significantly more fledglings than those breeding in nest boxes on utility poles (Table 2), with the former producing approximately three times more fledglings than the latter (Figure 3C).

**Figure 4:**
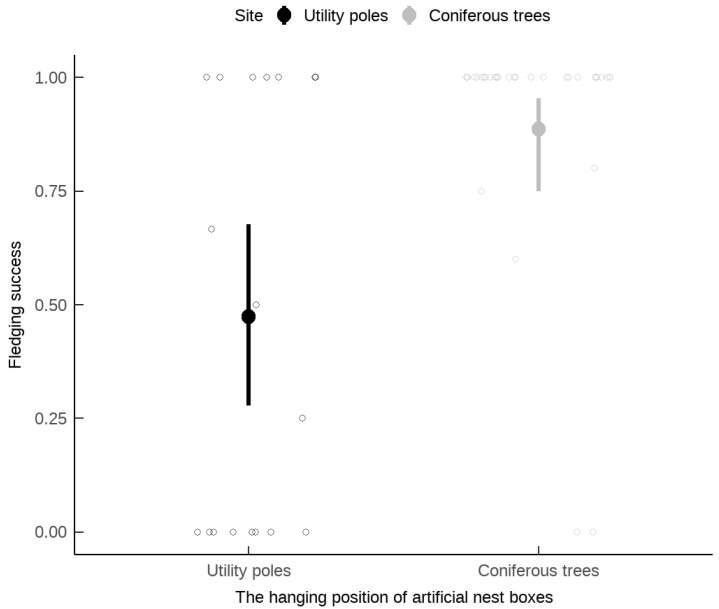
The effect of the tree species where nest boxes were hung on fledging success of *Passer cinnamomeus*. The points in the figure represent the jittered original data. The solid circles and vertical lines represent the average success rate and the 95% confidence interval of this type of tree species.

The fledging success of the russet sparrow was not significantly influenced by the distance of nest boxes to the nearest road, relative laying date, or brood size (Table 2). However, nests in boxes on coniferous trees had significantly higher fledging success compared to those in boxes on utility poles (Table 2 and Figure 4).

## 4. Discussion

This study found that the clutch size of the russet sparrow population mostly ranged from 4 to 5 eggs, with an average hatching rate of 86%. This is similar to the natural observations and descriptions of the breeding ecology of the russet sparrow in other regions such as Shanxi [24], Shaanxi, and Gansu [25], China. However, the fledging success was higher than in other studies, indicating that the russet sparrow can effectively utilize and adapt to artificial nest boxes for breeding. In addition, studies in the Kuankuoshui Nature Reserve in Guizhou have revealed that russet sparrow populations at different elevations exhibit variation in both laying dates and clutch size [26]. The russet sparrow population in this study initiated egg-laying earlier and had larger clutch sizes compared to the population at higher elevations in the Kuankuoshui Nature Reserve in Guizhou.

The most significant finding of this study is the observed association between the hanging position of nest boxes and reproductive success in russet sparrows. In this study, the nests of russet sparrows in the nest boxes hung at a further distance from the nearest road had a larger number of fledglings. This phenomenon may be attributed to the fact that roads can exert an influence on avian reproduction. For instance, issues such as habitat fragmentation and noise caused by roads are unfavorable for bird breeding [27]. This may be the reason that russet sparrows breeding in nest boxes closer to roads tended to have a smaller number of fledglings. The impact of nest box proximity to roads on avian reproductive success has been documented in other studies as well. For instance, the nesting success of white-crowned sparrow (*Zonotrichia leucophrys*) was found to be lower in nests closer to roads [28]. The breeding success of European pied flycatchers (*Ficedula hypoleuca*) during the nestling stage was more likely to fail in nest boxes near roads than in those further away, and a significantly higher number of nests near roads experienced complete brood mortality [29].

Also, our research indicated that the nests of russet sparrows in nest boxes on coniferous trees had significantly higher fledgling numbers and fledging success compared to those in nest boxes on utility poles, with the former producing nearly three times as many fledglings as the latter. This discrepancy may be attributed to the fact that nest boxes suspended from utility poles are mostly located along roadsides without shading, directly exposed to sunlight, and subject to greater human disturbance. Moreover, higher levels of human disturbance may also affect the feeding rate of parent birds, thereby influencing the development of nestlings. In contrast, nest boxes on coniferous trees typically offer greater shading and more stable environmental conditions, with less human disturbance, which may be more conducive to the breeding of the russet sparrow. Of course, this hypothesis warrants further verification in future studies. Furthermore, research has demonstrated that the tree species on which artificial nest boxes are suspended can influence avian reproductive success. For example, great tits (*Parus major*) breeding in nest boxes in deciduous forests initiate egg-laying earlier and have larger clutch sizes, whereas those breeding in nest boxes in coniferous forests produce more fledglings and have higher fledging success [30]. Thus, it is evident that the hanging position of nest boxes may exert a significant influence on avian reproduction.

It should be noted, however, that the observed variations in reproductive success may also relate to intrinsic differences in individual bird quality. Larger body size, better physical condition, or greater breeding experience could enhance competitive ability [31], allowing superior individuals to secure optimally positioned nest boxes (e.g., those further from roads or on coniferous trees) while simultaneously achieving higher reproductive output through enhanced parental care or resource acquisition. Future studies should quantify how individual quality mediates these relationships.

In addition, we found that russet sparrows laying eggs earlier had larger clutch sizes and brood sizes, as well as higher hatching rates in our study. Studies on other bird species have shown that individuals breeding earlier can invest more energy in reproduction [32]. Also, earlier-breeding females, when in better physical condition, are better able to balance their own physiological needs with the demands of parental care for offspring [33]. This allows them to lay higher quality or larger clutches of eggs and invest more effort into incubation, resulting in higher hatching success rates and larger brood sizes. Conversely, nests with later laying dates likely belong to individuals of poorer quality or those that have already undergone one breeding attempt, resulting in a decline in their physical condition. This decline subsequently reduces the quality or quantity of eggs laid and the effort invested in incubation, leading to decreased brood size and hatching rate. Exact reasons for the patterns found in russet sparrows may require further research.

## 5. Conclusions

This study not only enriches existing data on russet sparrows breeding in artificial nest boxes but also reveals an association between nest box hanging position and reproductive success. Recently, the potential adverse effects of artificial nest boxes on attracted bird species have gained increasing attention [17]. As a case study on the relationship of nest box placement with avian reproductive success, our findings underscore the importance of carefully considering hanging positions when deploying artificial nest boxes for bird attraction, ensuring optimal breeding outcomes for target species. Particularly significant is the widespread application of artificial nest boxes in the conservation of rare and endangered bird species, such as the wreathed hornbill (*Rhyticeros undulatus*) [34], lesser kestrel (*Falco naumanni*) [35], and swift parrot (*Lathamus discolor*) [36]. Timely understanding of the correlation between nest box hanging position and bird breeding and selecting appropriate locations for nest box installation are undoubtedly extremely important for improving conservation effectiveness.

## Figures and Tables

**Table 1:** Breeding parameters of *Passer cinnamomeus*.

Breeding Parameters	Sample Size	Minimum	Maximum	Mean	SD	Coefficient of Variation
Clutch size	64	3	6	4.61	0.58	0.13
Incubation period (d)	18	8	13	11.38	1.24	0.11
Brood size	57	2	6	3.93	0.94	0.24
Hatching rate	57	0.4	1	0.86	0.18	0.21
Number of fledglings	44	1	6	3.68	1.09	0.30
Fledging success	44	0.25	1	0.93	0.18	0.19

**Table 2:** Results of model analyses of the factors affecting the reproductive success of *Passer cinnamomeus*.

Breeding Parameters	Explanatory Variables	Estimate	SE	*Z*	*p*
Clutch size	Intercept	4.83	0.12	40.21	<0.001
	Relative laying date	−0.009	0.004	−2.34	0.020
Brood size	Intercept	4.26	0.26	16.23	<0.001
	Relative laying date	−0.02	0.008	−2.77	0.006
Hatching rate	Intercept	6.17	1.84	3.36	<0.001
	Relative laying date	−0.03	0.01	−3.07	0.002
	Clutch size	−0.77	0.35	−2.19	0.029
Number of fledglings	Intercept	−1.29	0.76	−1.69	0.091
	Distance from the nest site to the nearest road	−2.76	1.58	−1.74	0.081
	(Distance from the nest site to the nearest road)^2^	3.94	1.25	3.15	0.002
	Brood size	0.75	0.19	3.84	<0.001
	Coniferous trees ^a^	2.09	0.47	4.44	<0.001
Fledging success	Intercept	−0.10	0.43	−0.24	0.811
	Coniferous trees ^a^	2.17	0.66	3.30	<0.001

The “a” represents the parameter estimation of coniferous trees relative to utility poles.

## Data Availability

The raw data supporting the conclusions of this article will be made available by the authors on request.
